# TPPS4 inhibits PEDV by stabilizing viral RNA G-quadruplex and promoting ER stress: a transfer-learning-driven discovery

**DOI:** 10.1128/jvi.00774-26

**Published:** 2026-07-27

**Authors:** Yingge Zheng, Dehua Luo, Qingyan Tian, Mengdi Zhang, Yanfei Zhang, Yijia Zhang, Yuxuan Xu, Jingyi Wang, Yuxiang Wang, Wanjiang Ai, Hui Song, Wentao Li, Dengguo Wei

**Affiliations:** 1 State Key Laboratory of Agricultural Microbiology, College of Veterinary Medicine, Huazhong Agricultural University 47895, Wuhan, Hubei, China; 2 Hubei Hongshan Laboratory, Huazhong Agricultural University 47895, Wuhan, Hubei, China; 3 National Reference Laboratory of Veterinary Drug Residues (HZAU) and MAO Key Laboratory for Detection of Veterinary Drug Residues, Huazhong Agricultural University 47895, Wuhan, Hubei, China; 4 Shenzhen Branch, Genome Analysis Laboratory of the Ministry, Huazhong Agricultural University 47895, Shenzhen, China; 5 Shenzhen Institute of Nutrition and Health, Huazhong Agricultural University, Shenzhen, China; University of Minnesota Twin Cities, Minneapolis, Minnesota, USA

**Keywords:** PEDV, TPPS4, G-quadruplex, transfer learning

## Abstract

Porcine epidemic diarrhea virus (PEDV) is a devastating enteric pathogen that causes substantial economic losses in the global swine industry. While PEDV inhibitors offer a promising alternative to compensate for vaccine evasion caused by viral mutations, their development is bottlenecked by poorly defined antiviral targets and limited compound libraries. Here, we developed a transfer learning framework to accelerate the discovery of anti-PEDV agents by leveraging data from human-associated coronaviruses. Transfer learning-based prediction identified Hemin as a promising PEDV inhibitor, while its analog TPPS4 exhibited potent antiviral activity with EC_50_ values of 0.85 and 2.86 μM in Vero and LLC-PK1 cells, respectively. Notably, *in vivo* oral TPPS4 treatment lowered intestinal PEDV loads and doubled the piglet survival rate. Mechanistically, TPPS4 suppresses viral replication by stabilizing highly conserved G-quadruplex structures within the viral ORF1ab gene and simultaneously modulating host endoplasmic reticulum (ER) stress. This study demonstrates that transfer learning driven by human drug data facilitates the discovery of veterinary agents with diverse mechanisms of action, offering a novel paradigm for the development of other new veterinary therapeutics.

**IMPORTANCE:**

Veterinary antiviral discovery is hampered by limited bioactivity data and poorly defined targets. To address this, we developed a cross-species transfer learning pipeline to predict anti-PEDV agents. We identified TPPS4 as a potent anti-PEDV compound *in vitro* and *in vivo*, which exerts antiviral activity by stabilizing viral G-quadruplex structures and inducing host ER stress. This work establishes a workflow from computational screening to *in vivo* efficacy validation, demonstrating that cross-species transfer learning can accelerate veterinary antiviral discovery.

## INTRODUCTION

Swine production is a cornerstone of global animal husbandry and an essential component of the food supply chain. However, the persistent outbreaks of porcine epidemic diarrhea virus (PEDV) have inflicted devastating economic losses on the agricultural sector worldwide (1, 2). The rapid mutation of viral strains results in the vaccines not providing complete protection. This creates an urgent need for antiviral drugs to navigate the critical window period (3, 4). Conventional drug computational screening methods require accurate target protein structures and extensive annotated chemical libraries. This is prevented by inadequate understanding of animal virus-host interactions, incomplete structural data, and insufficient characterization of binding sites (5–7). These constraints highlight the need for more adaptable computational techniques.

Transfer learning, a sophisticated branch of machine learning, offers a powerful paradigm to overcome this bottleneck by leveraging knowledge from data-abundant domains to data-scarce ones, enabling cross-task generalization of model performance (8–14). The activity prediction accuracy of this approach will be improved by over 40% under small-sample conditions (15, 16). Transfer learning leverages biological data from the source domain to bypass resolution limitations of structural data in the target domain, thereby providing an efficient and scalable computational framework for drug screening and multi-target drug discovery (17).

In this study, we employed a cross-species transfer learning strategy to mitigate the data scarcity in veterinary antiviral research. We developed a predictive model, PEDV-TL, by fine-tuning a pre-trained framework originally constructed on human coronavirus inhibitors. This model successfully identified the porphyrin compound Hemin as a top candidate against the PEDV. Furthermore, TPPS4, an analog of Hemin, effectively inhibited multiple stages of PEDV proliferation via different mechanisms, which involved stabilizing G-quadruplex structures in PEDV genome and promoting ER stress. By integrating AI-driven chemical screening with mechanistic validation and *in vivo* trials, this work establishes a highly efficient paradigm for the discovery of veterinary chemical leads to safeguard animal-derived food production.

## MATERIALS AND METHODS

### Data

For the Human-COVID set, antiviral compounds against SARS, SARS-CoV-2, MERS, HCoV-OC43, and other related human coronaviruses were collected and integrated as source data sets (18, 19). The data set contained 420 positive data and 1,737 negative data. Compounds were classified as positive if they met the following criteria: (i) IC_50_ or EC_50_ < 10 µM, or EC_50_ > 50% inhibition at 10 μM, and (ii) demonstrated efficacy against at least one human coronavirus. All remaining compounds were classified as negative.

For the PEDV set, PEDV antiviral inhibitors containing 139 positive data and 187 negative data were collected as the target data set (20, 21). The data classification criteria are consistent with Human-COVID set.

For the screened set, natural products and their derivatives from SuperNatural III database were collected to construct a data set containing 6,898 compounds (22).

For the screening set 2, 19 porphyrin derivatives were added to the screened set.

### Transfer learning strategy

For construction of COVIDVS, the source model was built based on the framework proposed by Wang (19). This model leveraged a directed message passing neural network (D-MPNN) to learn molecular representations by capturing edge-specific features from directed graphs. These representations were subsequently processed through a three-layer feed-forward neural network to predict bioactivity. To ensure predictive stability and avoid overfitting, we employed an ensemble learning strategy using independent sub-models initialized with distinct random seeds. Hyperparameters were optimized via Bayesian optimization for 20 iterations. Each iteration adopted a unique random 8:1:1 train-validation-test split of the Human-COVID data set. The source model COVIDVS was first pre-trained on the Human-COVID data set, with model performance evaluated using the ROC-AUC metric.

For fine-tuning for PEDV (PEDV-TL), to address the data shortage in veterinary medicine, we adapted the COVIDVS model to the PEDV domain via a stratified fine-tuning strategy. Critical low-level feature extraction layers were frozen to prevent catastrophic forgetting, while higher-level parameters, comprising the three-layer feedforward network, remained trainable to capture task-specific patterns. Fine-tuning was performed using the Adam optimizer with a dynamic learning rate scheduler on a partitioned target data set (split into training, validation, and test sets in an 8:1:1 ratio). To ensure a comprehensive evaluation, particularly given the potential for class imbalance, the final model’s performance was rigorously assessed with accuracy, precision, recall, and F1-score.

### Cells, virus, and reagents

Cell lines used in this study included Vero E6 (ATCC, CCL-81), LLC-PK1 (ATCC, CL-101), and L929 (ATCC, CCL-1). All cells were cultured in medium (Dulbecco’s modified Eagle medium, Gibco) supplemented with 10% FBS at 37°C in 5% CO_2_. PEDV AJ1102 (GenBank: MK584552.1), MHV A59 (GenBank: MF618253.1), and PDCoV CHN-HN-2014 (GenBank: KT336560.1) were propagated in DMEM, supplemented with 7.5 μg/mL trypsin for PEDV and PDCoV or 2% FBS for MHV A59. All strains were kindly provided by Prof. Shaobo Xiao, Huazhong Agricultural University. Anti-PEDV N protein antibody was stored in our laboratory. Nsp14 antibody was kindly provided by Prof. Boli Hu of Zhejiang University. Other antibodies were purchased from Proteintech Group. The compounds were commercially obtained from Macklin, dissolved in dimethyl sulfoxide.

### Cytotoxicity assay

Cells in 96-well plates were exposed toTPPS4 (0–100 µM) for 24 h. Then, cell viability was measured by Cell Counting Kit-8 (Beyotime, Shanghai, China).

### Viral titration assay

Cells in 24-well plates were pretreated with TPPS4 before inoculation with different viruses. After a 2-h infection (PEDV: 0.1 MOI; MHV, PDCoV, and PoRV: 0.5 MOI), the medium was replaced with compound-containing medium for 18 h. Lysates were collected for viral titer determination by TCID_50_.

### Indirect immunofluorescence assay (IFA)

Following viral infection and compound treatment, cells were incubated first with PEDV N protein antibody after fixation with 4% paraformaldehyde (Biosharp, Beijing, China), and then with an Alexa Fluor 488-conjugated secondary antibody (Abbkine, Wuhan, China). After counterstaining the nuclei with DAPI (Solarbio, Beijing, China), the samples were imaged on a fluorescence microscope (Olympus, BX63).

### RNA isolation and quantitative real-time PCR (RT-qPCR)

Total RNA was reverse transcribed into cDNA using HiScript II 1st Strand cDNA Synthesis Kit (Vazyme, Nanjing, China) after extraction using RNAiso Plus (TaKaRa, Beijing, China). RT-qPCR was performed with SYBR qPCR Master Mix (Vazyme, Nanjing, China) and the primers specified in Table S1.

### Inhibitory activity of compounds at different stages of viral proliferation

For the attachment assay, Vero cells were cooled to 4°C and incubated with the mixture of PEDV and TPPS4 (5–40 µM) for 1 h. After washing with PBS, cells were maintained in culture until 18 h post-infection (hpi). For the internalization assay, cells were inoculated with PEDV at 4°C for 1 h, followed by a 2-h incubation with TPPS4 at 37°C before incubating with fresh medium. For the replication assay, following PEDV infection for 2 h at 37°C, cells were washed and continuously cultured in compound-containing medium until 16 hpi. For the release assay, at 16 hpi, the cells were washed, overlaid with fresh compound-containing medium, and supernatant samples were collected at 15, 30, and 60 min post-treatment for viral titration. For the inactivation assay, PEDV was pre-incubated with TPPS4 at 37°C for 2 h. The mixture was added to cells for 1 h, then replaced with fresh medium and cultured to 18 hpi. All samples at 18 hpi were collected for TCID_50_ assay.

### Western blot analysis

Cells were lysed, and the protein supernatant was collected and separated by SDS-PAGE (Epizyme Biotech). Proteins were incubated with primary antibodies for 4 h and then with secondary antibodies for 1 h. After washing, proteins were detected using ECL substrate (Vazyme, Nanjing, China) and visualized with a chemiluminescence imager (Tanon 5200).

### Animal experiment

For the acute toxicity test, SPF KM mice were randomly assigned to an administration group and a control group. Mice in the administration groups received a dose of 5,000 mg/kg orally. Clinical signs and body weights were recorded daily for 14 days. Data were from two independent replicate experiments.

For the *in vivo* antiviral activity assay of TPPS4, according to the 3R principle, 15 5-day-old piglets were allocated into three groups (*n* = 5) at random, comprising a mock group, a PEDV-infected group (orally inoculated with 10^5^ TCID_50_ PEDV), and a TPPS4 treatment group (orally inoculated with PEDV and treated with 30 mg/kg TPPS4). The treatment regimen began 24 h prior to viral infection and was administered daily until day 10 post-infection. At the study endpoint, euthanasia of piglets was carried out in adherence to institutional ethical standards for animal research. Viral loads of tissues and anal swabs were subsequently quantified.

### Clinical evaluation and fecal evaluation

Piglets were subjected to daily observation and evaluated for clinical symptoms and fecal consistency scores. Clinical symptoms were assessed on a scale from 0 to 3, where 0 indicated normal condition, and three represented the most severe clinical manifestations. Fecal consistency was scored from 0 to 3, with 0 for normal stools and 3 for watery diarrhea. Death of a piglet was assigned the score of 4, as the most severe endpoint.

### Hematoxylin-eosin staining (H&E stain)

The intestinal tissues of the piglets were collected after the experiment. After the jejunal tissue was fixed and embedded, tissue sections were prepared and stained with H&E for microscopic evaluation.

### Analysis of RNA-seq

Cells following PEDV infection, with or without subsequent TPPS4 treatment, were lysed for RNA extraction, after which mRNA was purified from the total RNA. Sequencing libraries were constructed and sequenced paired-end on an Illumina NovaSeq 6000 (Illumina). Raw sequencing data were quality-controlled and filtered with Fastp. The quality control data were respectively aligned to the reference genome of *Chlorocebus sabaeus* and PEDV AJ1102 strain genome by STAR align (version 2.7.10a). For the analysis of host gene expression, the R package DESeq2 was used for differential expression analysis. The differentially expressed gene was defined as the adjusted *P*-value after error detection rate (FDR) correction < 0.05. The differentially expressed genes were subjected to Gene Ontology (GO) enrichment analysis. The transcriptional level of each ORF, relative to the full-length genomic reads, was compared between the PEDV group and the TPPS4-treated group.

### Circular dichroism (CD) spectroscopy and CD melting assays

RNA was dissolved in buffer with different KCl concentrations (0, 50, 100 mM). Spectra were collected at 25°C using the JASCO-810 spectrophotometer (1.0-mm optical path quartz cell). The scanning range was 200-320 nm. All CD spectra were corrected by subtracting the buffer baseline. TPPS4 was added to RNA solution (20 μM). After heating the sample at 95°C for 10 min and cooling it gradually, the melting curves were recorded using JASCO-810 with a temperature range of 4.0°C–98.0°C, and with an interval of 1°C.

### Nuclear magnetic resonance (NMR) spectroscopy

For NMR analysis, 0.5-mM RNA samples were prepared in a DEPC-treated buffer (200 mM KCl, 10% D₂O), thermally annealed (heated to 95°C for 5 min and slowly cooled to 25°C), and then analyzed at 298 K on an 800 MHz Bruker Avance DRX-800 spectrometer. Instrument parameters included: diffusion time (TD) = 100 ms, eddy current recovery time (TE) = 50 ms, and relaxation delay (TR) = 2 s.

### RNA stop assay

The mixture of P15 RNA primer and template RNA underwent thermal annealing at 95°C for 5.0 min, followed by gradual cooling to 4°C. Subsequently, nucleoside triphosphates and recombinant RNA-dependent RNA polymerase were added into the reaction mixture for 20 minutes at 33°C. Reactions were terminated by adding 95% formamide and heating to 90°C for 10 min for denaturation. The denatured products were then separated on a 20% denaturing polyacrylamide gel and visualized by fluorescence scanning using a Pharos FX Molecular Imager (Bio-Rad).

### EGFP reporter assay

The PQSnsp14 or PQSnsp14mut sequence was cloned into the N-terminus of EGFP in pEGFP-C1 vector. Vero cells (60%–70% confluence in 24-well plates) were transfected with the vector, pEGFP-PQSnsp14, or pEGFP-PQSnsp14mut using Lipofectamine 3000 (Thermo Fisher Scientific). At 6 h post-transfection, cells were treated with TPPS4 (0, 5, 10, 20 μM) for 36 h. Cells were then washed with PBS, fixed with 4% paraformaldehyde for 15 min at room temperature, and permeabilized with ice-cold methanol for 10 min. After washing with PBS, nuclei were stained with DAPI (Solarbio) for 15 min and then imaged via a two-photon laser scanning confocal microscope (Olympus FV1000MP).

### Construction of the recombinant strain rPEDV-PQSnsp14mut

According to the method ofLuo for constructing recombinant strains, G-rich region (17720-17734nt, PQSnsp14) was mutated to PQSnsp14mut (23). The PQSmut fragment was obtained by overlap PCR on the products of two pairs of primers PQSmutF1/R1 and PQSmutF2/R2. Guide RNA (sgRNA) was synthesized by *in vitro* transcription from a DNA template, which was amplified using scaffold oligo with primers PQSmut-sgRNA-a and PQSmut-sgRNA-b. The pBAC-CMV-PEDV plasmid was digested with Cas9/sgRNA, and the PQSmut fragment was inserted to generate pBAC-PEDV-PQSmut. This plasmid was transfected into Vero cells using Lipofectamine 3000 (Thermo, L3000015) until the recombinant mutant virus (rPEDV-PQSnsp14mut) was obtained and confirmed by Sanger sequencing successfully. The primer sequences are shown in Table S1.

### Statistical analysis

Statistical significance (*P* < 0.05) was assessed by Student’s *t*-test, and one-way or two-way ANOVA using GraphPad Prism 8.

## RESULTS

### Construction of anti-coronavirus inhibitor screening model

**Fig 1:**
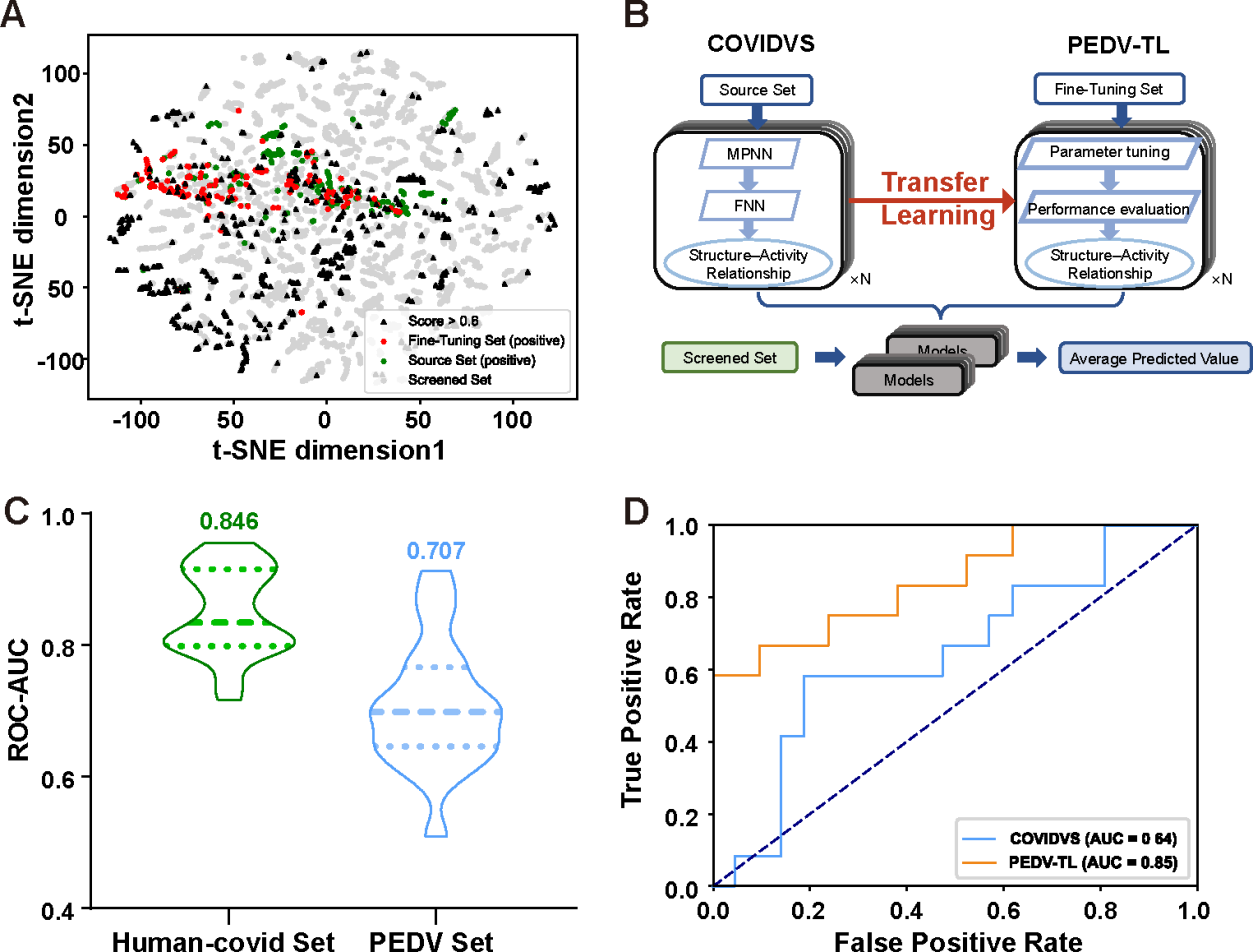
Construction of COVIDVS model and PEDV-TL model. (**A**) Chemical spatial distribution of Human-COVID set-positive (green), PEDV fine-tuning set-positive (red), screened set (gray), score > 0.6 of screened set (black). (**B**) Diagram of the generation process of COVIDVS and PEDV-TL. (**C**) AUC of the COVIDVS ensemble (20 models) on a 10% test set from the Human-COVID training data (green) and the PEDV set (blue). (**D**) ROC curves of PEDV-TL and COVIDVS models in distinguishing active and inactive data.

The Human-COVID set was compiled, containing 420 active and 1,737 inactive entries of inhibitors against human coronaviruses (HCoV-OC43, SARS-CoV, MERS-CoV, SARS-CoV-2, and others). *t*-distributed stochastic neighbor embedding (t-SNE) visualization demonstrated the structural diversity of compounds within the Human-COVID set in chemical space. (Fig. 1A).

A general anti-coronavirus inhibitor classification model named COVIDVS was trained based on the Human-COVID set. Built on the Chemprop framework, COVIDVS combines a message-passing neural network (MPNN) module for molecular feature extraction with a feedforward neural network (FNN) classification module (Fig. 1B) (19, 24). The COVIDVS model achieved an average ROC-AUC of 0.846 on its corresponding 10% test subsets (Fig. 1C), demonstrating robust performance in distinguishing active from inactive compounds within the Human-COVID set.

### Generation of the PEDV-TL model via fine-tuning COVIDVS

Furthermore, we compiled a data set of PEDV inhibitors from the literature, comprising 139 active and 187 inactive compounds, named PEDV Set. This data set is non-redundant with the Human-covid Set. The moderate ROC-AUC value of 0.707 for COVIDVS on the PEDV set indicates limited generalization ability toward screening PEDV inhibitors (Fig. 1C). Consequently, transfer learning optimization for the COVIDVS model is essential to bridge this domain discrepancy (Fig. 1B).

The PEDV set was randomly divided into the fine-tuning data set (PEDV fine-tuning set) and the independent test set (PEDV test set) in a ratio of 9:1. The PEDV-TL model was developed by fine-tuning parameters of COVIDVS based on the PEDV fine-tuning set, thereby preserving transferable features learned from the source domain while adapting to target-specific characteristics (Fig. 1B). To prevent overfitting on limited data, we froze the cached_zero_vector and W_i.weight parameters in the MPNN module to maintain their core functionality, while the remaining MPNN components and the classification module were fine-tuned. PEDV-TL, an ensemble of 20 fine-tuned COVIDVS base models, achieved an ROC-AUC of 0.850 on the PEDV test set, significantly outperforming the original COVIDVS (Fig. 1D). Moreover, the PEDV-TL model achieved excellent performance across all evaluation metrics on the PEDV test set, demonstrating high generalization capability and stability (Table 1), which validated the efficacy of the transfer learning approach.

**TABLE 1:** Performance metrics of models COVIDVS and PEDV-TL on the PEDV test set^*a*^

Model	ROC-AUC	ACC	MCC	BA	F1
COVIDVS	0.643	0.606	−0.134	0.476	N/A
PEDV-TL	0.850	0.818	0.598	0.786	0.727

^*a*^ F1 score for COVIDVS is not applicable (N/A), as no true PEDV positives were detected.

### Activity prediction and validation of screened set based on the PEDV-TL model

The screened set consisted of 6,898 compounds, and the data set is primarily composed of natural products and their derivatives that we have collected from the literature and the SuperNatural III database (22). t-SNE dimensionality reduction was employed to visualize the chemical space distributions of the screened set alongside the Human-COVID set and PEDV fine-tuning set (Fig. 1A). Subsets of the screened set exhibited overlaps with positive compounds identified from the Human-covid and PEDV fine-tuning sets, indicating overlapping molecular bioactivity potential. Concurrently, the broad dispersion of the Screened Set across chemical space demonstrated substantial structural diversity, providing a robust foundation for inhibitors with novel structures.

**Fig 2:**
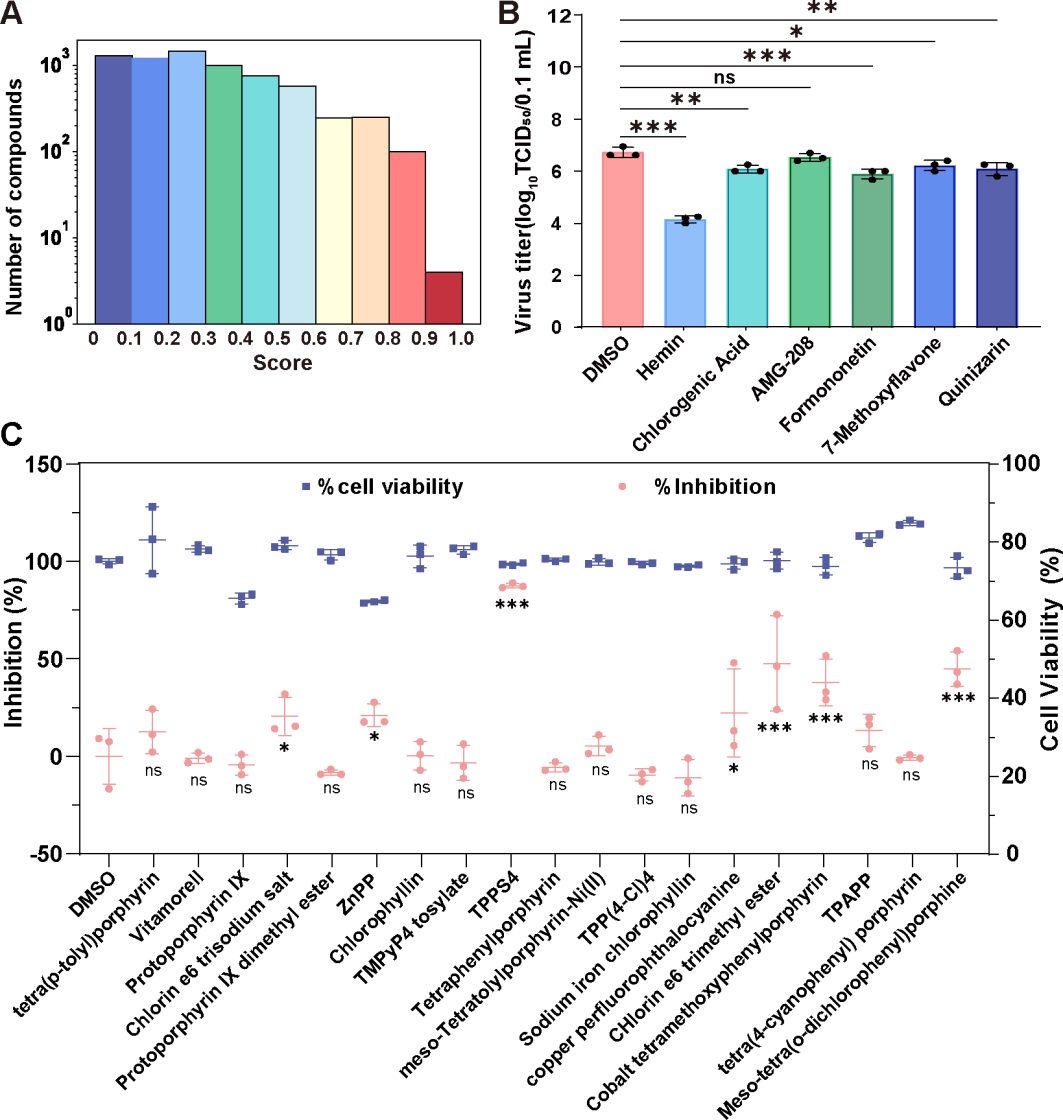
Antiviral activity of the Screened Set against PEDV. (**A**) Histogram of predicted score distribution of the screened set with the PEDV-TL model. (**B**) Anti-PEDV activity of 20-μM natural compounds measured by TCID_50_. (**C**) Cytotoxicity and antiviral activity assessment of porphyrin-scaffold compounds at 10 μM. Data are presented as mean and standard deviations (SDs) analyzed by one-way ANOVA from three independent experiments. Statistical significance is indicated by asterisks (*P < 0.05, **P < 0.01, ***P < 0.001, ns, not significant).

Predictive activity scores of compounds in the screened set, generated by the PEDV-TL model, revealed a distribution of predicted activity scores (Fig. 2A). Notably, wogonin, which has been previously documented as a PEDV inhibitor, achieved a score above 0.6 (25). This result validated the model’s reliability. To test the predictive accuracy, six compounds with scores above 0.6 in the prediction model were randomly selected for validation, which included hemin, AMG-208, chlorogenic acid, formononetin, quinizarin, and 7-methoxyflavone (Fig. 2B and Fig. S1). Crucially, hemin exhibited superior antiviral efficacy, resulting in an approximately 100-fold reduction in the viral titer (Fig. 2B).

**Fig 3:**
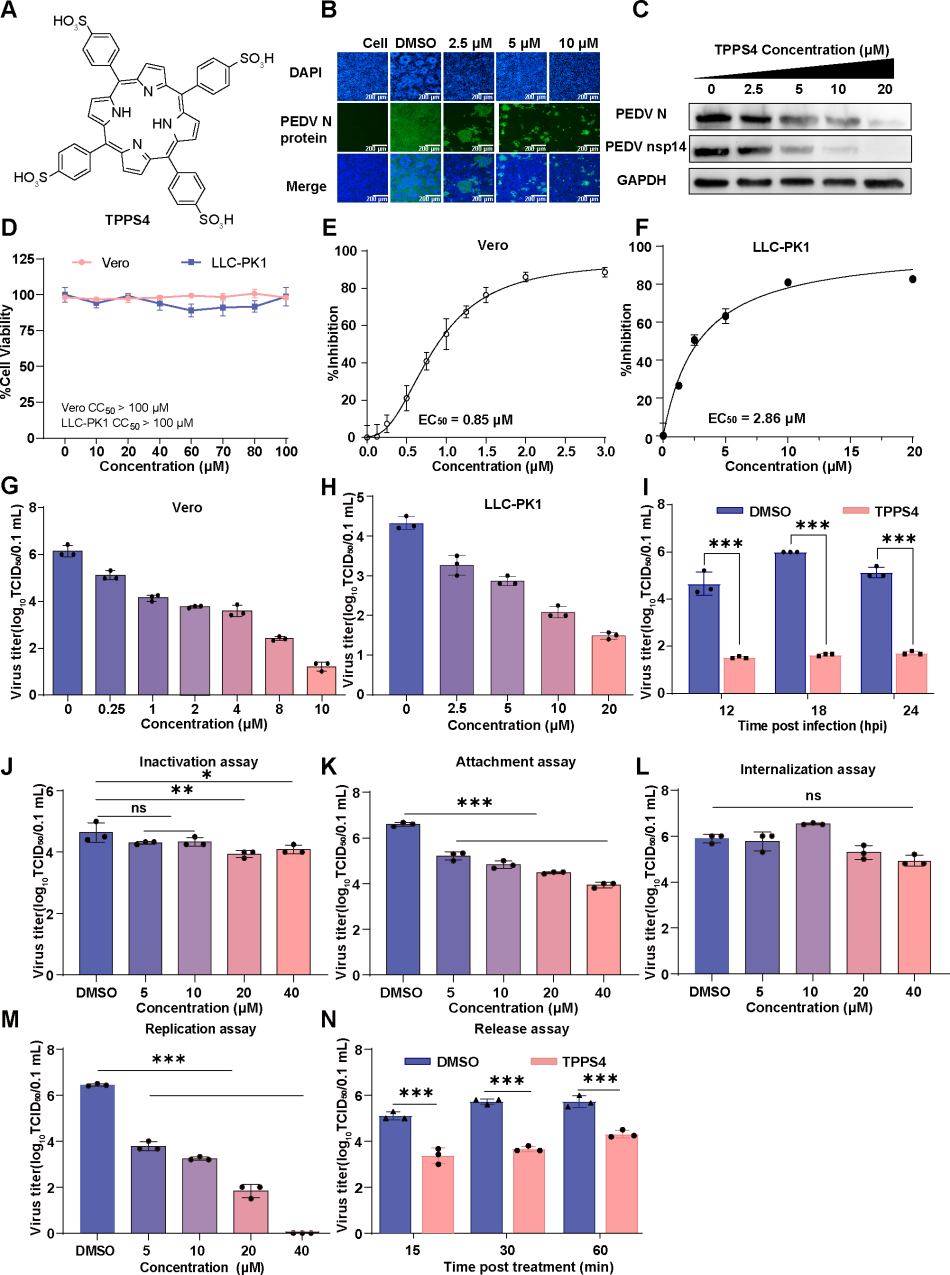
TPPS4 inhibited PEDV proliferation. (**A**) Structure of TPPS4. (**B and C**) The activity of TPPS4 against PEDV detected by IFA (**B**) and Western blot (**C**), scale bar: 200 μm. (**D**) CC_50_ of TPPS4 on Vero and LLC-PK1 cells detected by CCK-8 assay. (**E and F**) EC_50_ of TPPS4 against PEDV on Vero cells (**E**) and LLC-PK1 cells (**F**) detected by RT-qPCR. (**G and H**) Virus titer of TPPS4 against PEDV on Vero cells (**G**) and LLC-PK1 cells (**H**). (**I**) Time-dependent antiviral activity of 10 μM TPPS4 against PEDV. (**J–N**) Effects of TPPS4 on PEDV proliferation stages, inactivation assay (**J**), attachment assay (**K**), internalization assay (**L**), replication assay (**M**), and release assay (**N**). Data are mean ± SD of three independent experiments. One-way or two-way ANOVA was used (*P < 0.05, **P < 0.01, ***P < 0.001, ns: not significant).

A structural similarity search based on the porphyrin ring of hemin yielded 19 derivatives, designated screening set 2 for subsequent analysis (Fig. S2). Encouragingly, 13 of the 19 compounds ranked within the top 1% of predicted scores (score > 0.7), indicating antiviral potential. These analogs displayed generally low cytotoxicity and diverse levels of anti-PEDV activity, with TPPS4 showing the most potent inhibitory effect (Fig. 2C; Table S2). TPPS4 treatment induced a gradual reduction in PEDV N protein and nsp14 protein expression (Fig. 3A through C). Notably, TPPS4 exhibited half-maximal effective concentration (EC_50_) values of 0.85 μM in Vero cells and 2.86 μM in LLC-PK1 cells. Furthermore, the compound demonstrated low cytotoxicity (CC_50_ > 100 µM), resulting in a selectivity index (SI) significantly exceeding 10 (Fig. 3D through F).

Furthermore, TPPS4 reduced viral titers in a dose-dependent manner and exerted a sustained antiviral inhibition (Fig. 3G through I). TPPS4 simultaneously interfered with multiple critical stages of the PEDV life cycle, including viral attachment, replication, and release (Fig. 3J through N). Collectively, these results demonstrated that TPPS4 exerts anti-PEDV activity via pleiotropic regulatory mechanisms.

### *In vivo* protection of TPPS4 against PEDV infection

TPPS4 was proved to be practically nontoxic based on its minimal systemic toxicity. This classification was supported by findings in KM mice administered TPPS4 orally at 5,000 mg/kg, which showed progressive weight gain over 14 days and no obvious clinical symptoms throughout the observation period (Fig. S3).

**Fig 4:**
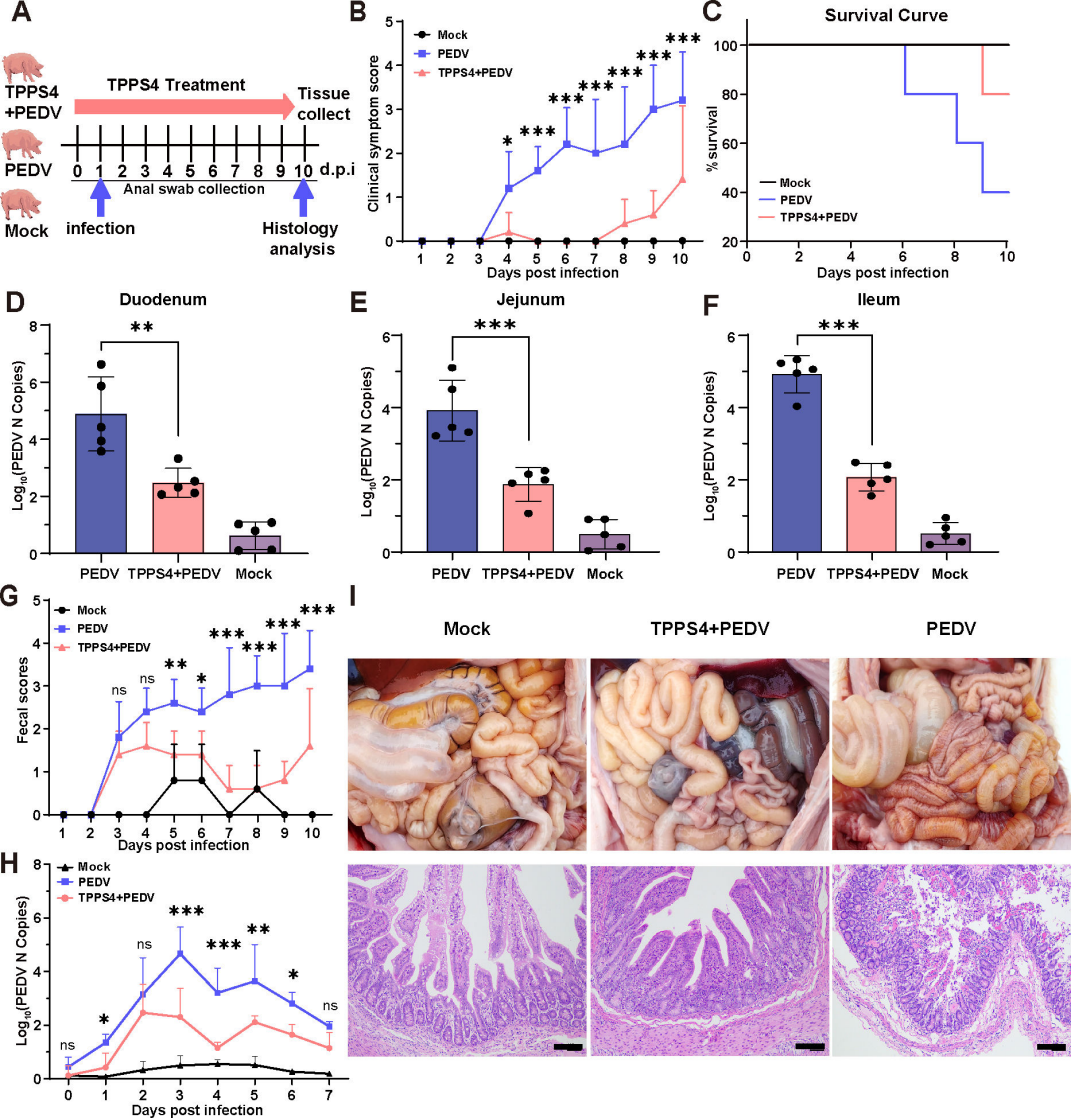
Inhibitory effect of TPPS4 on PEDV proliferation *in vivo*. (**A**) Schematic of animal experiment. Piglets (*n* = 5) of different groups were housed individually to prevent cross-infection. Piglets in the PEDV group and treatment group were orally inoculated with 10^5^ TCID_50_ of AJ1102 strain. Piglets in the treatment group received oral TPPS4 (30 mg/kg, dissolved in PBS) based on body weight daily. The mock group received the same volume of saline orally. (**B**) Clinical scores. (**C**) Survival curves. (**D–F**) Viral loads of duodenum (**D**), jejunum (**E**), and ileum (**F**). (**G**) Fecal scores. (**H**) Viral loads in anal swab. (**I**) Representative pathological image and H&E images of intestinal tissues, scale bar: 200 μm. Representative data were analyzed by one-way or two-way ANOVA (mean ± SD, *P < 0.05, **P < 0.01, ***P < 0.001, ns: not significant).

To evaluate *in vivo* antiviral efficacy, 5-day-old piglets (*n* = 5 per group) were divided into Mock group, PEDV group, and TPPS4 treatment group. Given the significance of prophylactic and early-stage treatment against PEDV infection, we focused on the prophylactic efficacy. Piglets in the treatment group received daily oral TPPS4 (30 mg/kg) commencing 24 h before inoculation with PEDV, with the PEDV group receiving saline (Fig. 4A). The PEDV group demonstrated severe symptoms, including acute vomiting, drowsiness, depression, watery diarrhea, gradual weight loss, and dehydration, while the clinical symptoms of the TPPS4 treatment group were relatively mild (Fig. 4B). At the end of the experiment, a survival rate of 80% was observed in the TPPS4 treatment group, compared with 40% in the PEDV group (Fig. 4C). Notably, viral loads in the duodenum, jejunum, and ileum were significantly reduced in the TPPS4-treated group relative to the PEDV group (Fig. 4D through F). The TPPS4 treatment group exhibited a relatively healthy fecal state and significantly reduced fecal viral RNA (Fig. 4G and H).

The intestines of the PEDV group exhibited pathological distension, congestive swelling, and thinning with translucency, whereas the TPPS4 treatment group maintained a relatively healthy architecture (Fig. 4I). Hematoxylin and eosin (H&E) staining revealed that the PEDV-infected group exhibited severe jejunal villous shedding, degeneration of intestinal mucosal epithelial cells, obvious inflammatory cell infiltration, and a marked reduction in goblet cells. In contrast, the TPPS4 treatment group maintained relatively intact jejunal villi and displayed remarkably alleviated inflammatory cell infiltration. These findings indicated that TPPS4 possessed significant efficacy in blocking PEDV proliferation *in vivo* and limiting viral transmission.

### TPPS4 stabilizes viral G-quadruplex structure and promotes host endoplasmic reticulum stress

**Fig 5:**
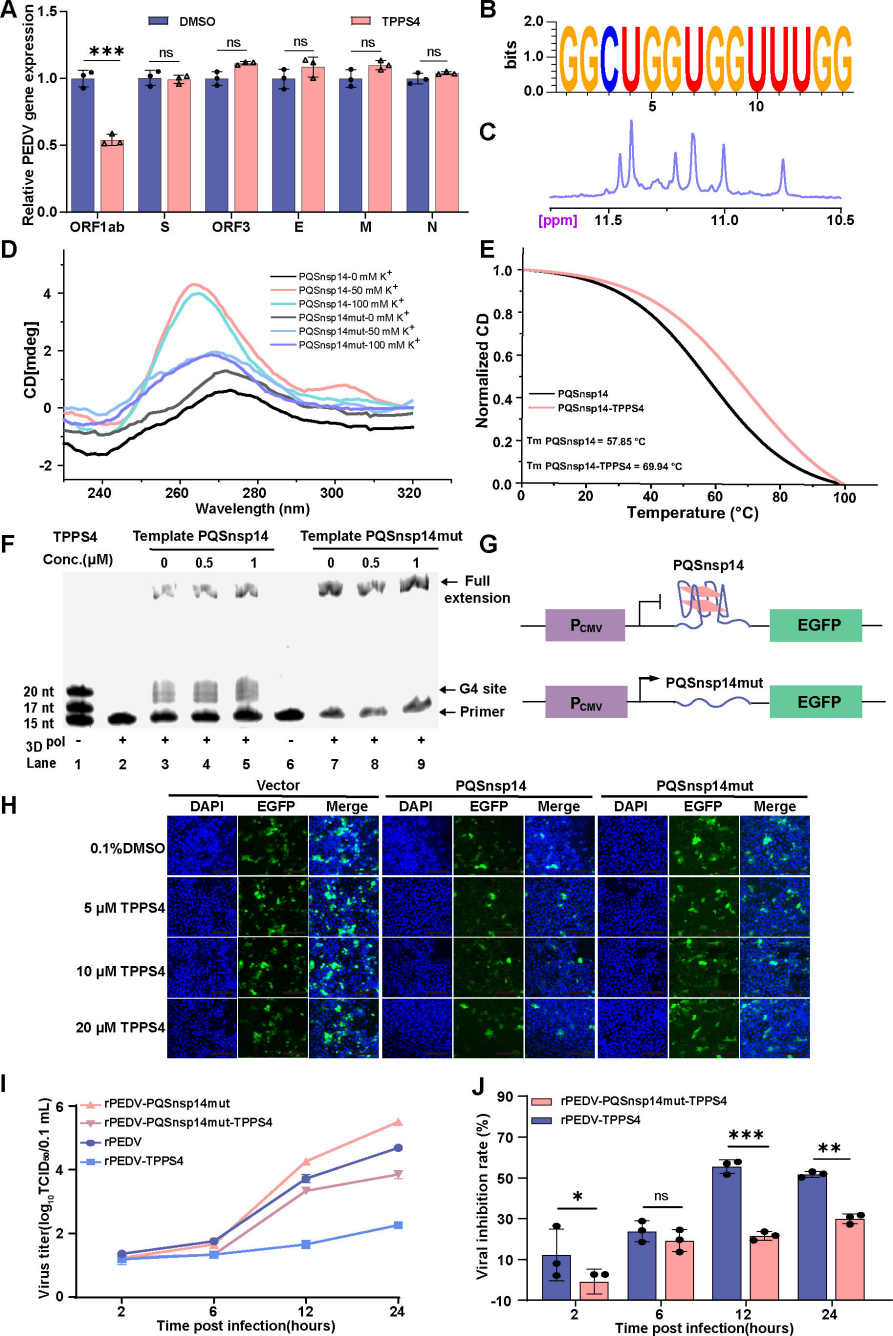
TPPS4 exerted antiviral activity by targeting PEDV G-quadruplex. (**A**) The effect of TPPS4 on the RNA abundance of PEDV ORFs. (**B**) Conservation of PQSnsp14 among different strains of PEDV is displayed by WebLogo, and the height of the letters represents the relative frequency of a specific nucleotide at that position. (**C**) NMR spectrum of PQSnsp14. (**D**) CD spectra of PQSnsp14 and PQSnsp14mut in solution with potassium at different concentrations. (**E**) CD melting curves of PQSnsp14 incubated with or without TPPS4. (**F**) RNA stop denaturing gel of PQSnsp14 and PQSnsp14mut incubated with TPPS4 at different concentrations. (**G**) Schematic of pEGFP-C1 reporter constructs. (**H**) Confocal micrographs showing EGFP expression in Vero cells. Scale bar: 100 μm. (**I**) Proliferation curves of rPEDV and rPEDV-PQSnsp14mut incubated with or without 10 μM TPPS4. (**J**) Viral inhibition rates of TPPS4 against rPEDV and rPEDV-PQSnsp14mut at different time points post-infection. Representative results are shown from three independent experiments. Statistical analysis was performed using two-way ANOVA (mean ± SD, *P < 0.05, **P < 0.01, ***P < 0.001, ns: not significant).

RNA-seq was performed to reveal the regulatory effects of TPPS4 on virus and host. The RNA level of each gene segment in the PEDV genome was quantified as the ratio of their average read numbers to that of the mRNA reads. RNA level of ORF1ab was specifically reduced with the addition of TPPS4, while other structural genes remained stable, suggesting its potential to downregulate ORF1ab transcription (Fig. 5A). This specific action of TPPS4 might involve targeting a unique RNA structure, which could be a target for drug development within ORF1ab RNA region (26–29). Notably, a conserved potential G-quadruplex-forming sequence (PQSnsp14: GGCUGGUGGUUUGG) was observed in PEDV ORF1ab, and NMR analysis confirmed its ability to form a G-quadruplex structure (Fig. 5B and C). In contrast, the mutant PQSnsp14mut (GGCUGGUGGUUUAG) fails to form the structure (Fig. 5D). CD melting assays showed that TPPS4 significantly increased the melting temperature (Tm) of PQSnsp14, indicating that TPPS4 could stabilize the G-quadruplex structure (Fig. 5E). RNA stop assay further showed that the G-quadruplex motif induced transcriptional arrest, and this inhibitory effect was further strengthened by TPPS4 treatment (Fig. 5F). In the EGFP reporter assay, the existence of PQSnsp14 significantly inhibited EGFP expression, and the addition of TPPS4 further enhanced this inhibitory effect. In contrast, PQSnsp14mut was not stabilized by TPPS4, resulting in negligible inhibition of EGFP expression (Fig. 5G and H). Collectively, these results suggest that the G-quadruplex motif triggers transcriptional arrest and consequently represses protein expression. As a G-quadruplex stabilizer, TPPS4 further reinforces this inhibitory effect on transcription and translation.

To determine whether PQSnsp14 serves as a viable antiviral target, we generated a recombinant PEDV strain carrying PQSnsp14mut sequence (rPEDV-PQSnsp14mut) using CRISPR-Cas9 technology. Growth curve analysis showed that rPEDV-PQSnsp14mut exhibited similar replication kinetics to rPEDV, with slightly faster proliferation after 12 hours post-infection (Fig. 5I). Besides, TPPS4 continuously inhibited rPEDV replication, achieving a 2.43 log10 TCID_50_ reduction at 24 hpi. In contrast, its effect on rPEDV-PQSnsp14mut was significantly weaker, with a 1.65 log10 TCID_50_ reduction (Fig. 5I). Taken together, PQSnsp14 may serve as a potential druggable target, and the selective antiviral activity of TPPS4 is likely mediated by stabilizing G-quadruplex structures in the viral genome. However, the inhibitory effect of TPPS4 on rPEDV-PQSnsp14mut did not completely vanish, suggesting that TPPS4 probably exerts its antiviral activity through additional mechanisms (Fig. 5I and J).

**Fig 6:**
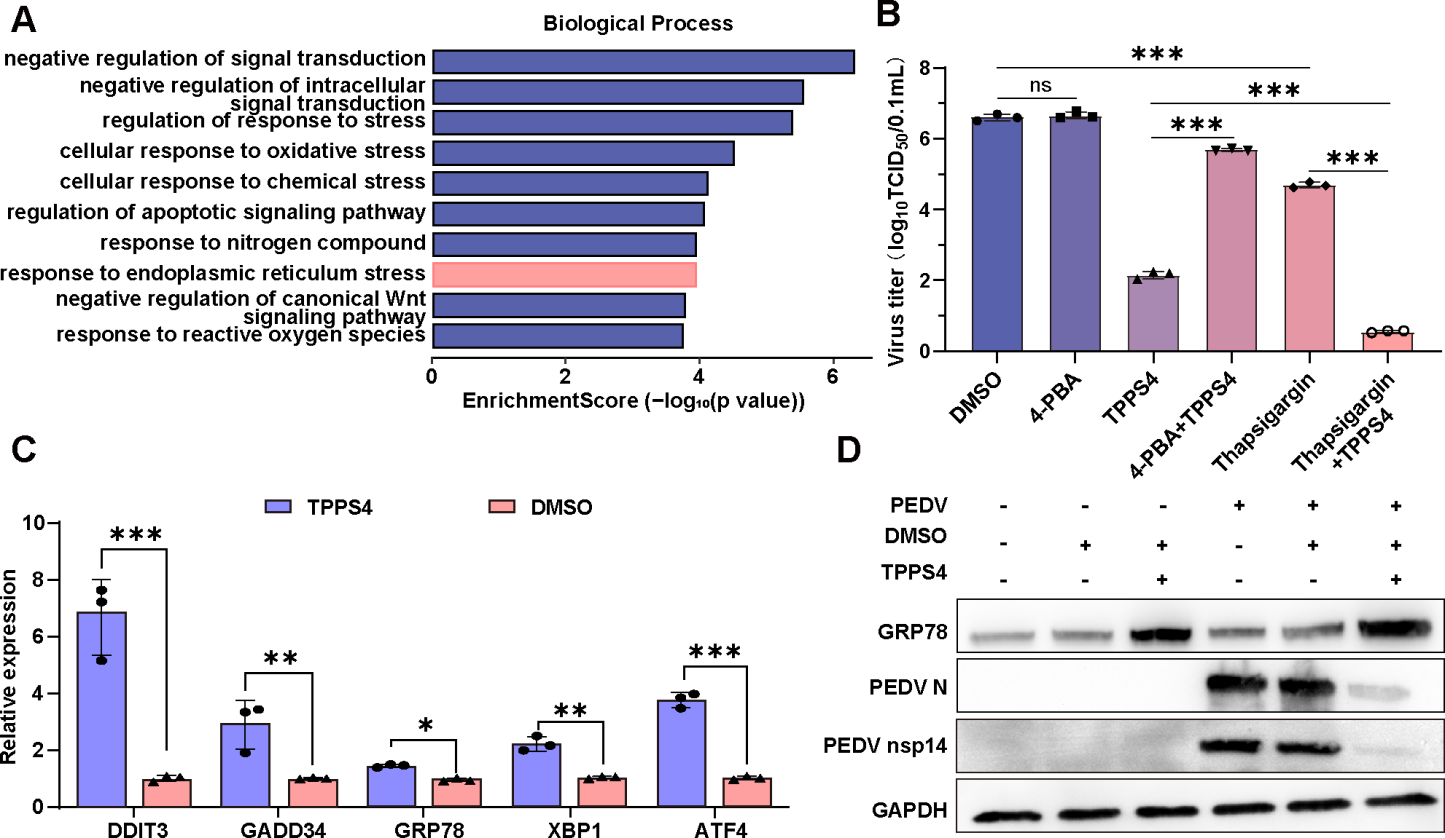
TPPS4 suppressed PEDV proliferation through ER stress induction. (**A**) GO results of RNA-seq for the host gene. GO analysis was performed on differentially expressed genes (DEGs) identified from a comparison between the TPPS4 treatment group and the PEDV infection group. (**B**) TCID_50_ assay of 4-PBA (0.3 mg/mL), TPPS4 (10 μM), 4-PBA (0.3 mg/mL) + TPPS4 (10 μM), thapsigargin (0.5 μM), thapsigargin (0.5 μM) + TPPS4 (10 μM) on PEDV inhibition. (**C**) RT-qPCR assay of ER stress-related genes. (**D**) Western blot analysis of GRP78, PEDV N protein, PEDV nsp14, and GAPDH expression. Representative data were analyzed by one-way ANOVA or two-tailed unpaired Student’s *t*-test from three independent experiments (mean ± SD, *P < 0.05, **P < 0.01, ***P < 0.001, ns: not significant).

RNA-seq and Gene Ontology (GO) enrichment analysis were performed to identify cellular pathways affected by TPPS4. The analysis revealed that TPPS4 regulates multiple biological processes, including negative regulation of signal transduction, cellular response to oxidative stress, ER stress response, and regulation of apoptotic signaling pathways (Fig. 6A). Consistent with reports that ER stress inhibits coronavirus proliferation (30, 31), the ER stress inducer thapsigargin significantly reduced PEDV replication (Fig. 6B). Notably, TPPS4 treatment significantly upregulated multiple ER stress-related genes, including DDIT3 (CHOP), GRP78, ATF4, XBP1, and GADD34 (Fig. 6C). The ER stress marker GRP78 was upregulated by TPPS4, regardless of the presence or absence of PEDV (Fig. 6D). Importantly, the antiviral activity of TPPS4 was partially antagonized by the ER stress inhibitor 4-phenylbutyric acid (4-PBA) and synergistically enhanced by the ER stress inducer thapsigargin (Fig. 6B). Collectively, these findings indicated that TPPS4 exerts its antiviral effect in part through induction of ER stress (31, 32).

## DISCUSSION

The development of efficient and safe veterinary interventions is paramount for the sustainability of the swine industry and the prevention of zoonotic risks. However, the discovery of novel chemical leads for agricultural pathogens often faces the “data desert” challenge. In this study, we successfully bridged the gap between data-rich human coronavirus research and data-scarce veterinary medicine by employing a transfer learning framework. Besides, we further verified that TPPS4 effectively inhibited the proliferation of Murine Hepatitis Virus (MHV) and Porcine deltacoronavirus (PDCoV), with EC_50_ values of 1.6 and 10.76 μM (Fig. S4). These findings firmly validate the broad-spectrum antiviral potency of TPPS4 and underscore the superiority of the transfer learning framework. This approach enables effective prediction and extrapolation of antiviral effects from well-studied human coronavirus systems to understudied animal viruses, offering a reliable strategy to accelerate the discovery and efficacy evaluation of broad-spectrum antiviral candidates in veterinary virology.

The identification of TPPS4 as a potent anti-PEDV lead highlights the potential of porphyrin derivatives in veterinary medicine. Porphyrins are structurally characterized by their macrocyclic scaffolds, which facilitate diverse biological interactions (33–35). The antiviral efficacy of TPPS4 indicates that its negative charge does not preclude cellular uptake, aligning with reports of cytoplasmic localization for TPPS4 and the cellular entry of anionic porphyrins via active mechanisms (36–38). This is corroborated by the cell-based antiviral activity of another anionic G-quadruplex ligand, NMM (39). TPPS4 inhibits viral replication by stabilizing G-quadruplex motifs. Additionally, it induces host ER stress, potentially priming cellular antiviral defenses (40). This dual-targeting mechanism is particularly advantageous in agriculture, as it may raise the genetic barrier to viral resistance. Crucially, the results of *in vivo* trials demonstrated a significant reduction in viral shedding and preservation of intestinal mucosal integrity in piglets (Fig. 4). These effects are key to limiting disease transmission and promoting growth in neonatal livestock, underscoring the translational potential for agricultural settings. Nevertheless, comprehensive *in vivo* safety and toxicological assessments remain essential to establish its agricultural suitability.

A deeper understanding of the divergences and commonalities between the source and target domains is essential to developing more precise and effective transfer learning approaches. Transfer learning shows broad potential in drug discovery tasks, including molecular property prediction, *de novo* compound generation, and synergy forecasting (17, 18, 41–44). This strategy is most effective when biological targets and signaling pathways are evolutionarily conserved across human and animal species. Even so, translating this theoretical concept into broad practical use remains challenging (15). The field is still emerging and must address several key issues: the absence of standardized evaluation frameworks, the risk of overfitting in data-scarce settings, limited model interpretability, and the threat of “negative transfer” arising from insufficient domain relevance or significant species divergence. Therefore, to fully leverage the rich data from human medical research for veterinary drug discovery, it is critical to systematically map both the similarities and distinctions between human and veterinary systems—from molecular targets to functional pathways. Through careful model fine-tuning and rigorous experimental validation, the vast knowledge accumulated in human medicine can be efficiently and safely translated into innovations for animal health, thereby supporting veterinary therapeutic development and broader public health security.

## Data Availability

The data underlying this article will be shared on reasonable request to the corresponding author. The source code is accessible via https://github.com/HZAU-Weilab/Transfer-Learning-model.

## SUPPLEMENTAL MATERIAL

Supplemental material jvi.00774-26-s0001.docx Fig. S1 to S4; Tables S1 and S2.

ASM does not own the copyrights to Supplemental Material that may be linked to, or accessed through, an article. The authors have granted ASM a non-exclusive, world-wide license to publish the Supplemental Material files. Please contact the corresponding author directly for reuse.
