# Supplementary material for: TPPS4 inhibits PEDV by stabilizing viral RNA G-quadruplex and promoting ER stress: a transfer-learning-driven discovery
Source: J Virol. 2026 Jul 27;100(8):e00774-26. doi: 10.1128/jvi.00774-26 (PMC13483440; doi:10.1128/jvi.00774-26)
Supplement: Supplementary file 1 [file jvi.00774-26-s0001.docx]

**Supplementary Material**

**TPPS4 Inhibits PEDV Replication by Stabilizing RNA G‑quadruplex and Promoting ER Stress: A Transfer‑Learning‑Guided Antiviral Discovery**

**Figure S1. Chemical structures of the natural products investigated in this study.**

**Figure S2. Structures of the compounds with a porphyrin ring used in this study.**

**Figure S3. TPPS4 exhibited negligible acute toxicity.**

Following a 3-5-day acclimation period, 4-week-old KM mice received oral TPPS4 administration (5000 mg/kg) with daily monitoring of body weight and clinical symptoms for 14 days. Body weight of male mice (A), and female mice (B).

**Figure S4. TPPS4 inhibited the proliferation of MHV and PDCoV.**

(A, B) Cell viability and antiviral activity of TPPS4 against MHV (A) and PDCoV (B). Inhibition was quantified by RT-qPCR. (C, D) Virus titers of TPPS4 against MHV (C) and PDCoV (D).

**Table S1. Primer sequences used in this article.**

| Primers | Sequence (5’- 3’) |
| --- | --- |
| qGAPDH-F | GATCATCAGCAATGCCTCCT |
| qGAPDH-R | TGAGTCCTTCCACGATACCA |
| qPEDV-F | CTGGGTTGCTAAAGAAGGCG |
| qPEDV-R | CTCGGGAGCTGTTGAGAGAA |
| qMHV-F | ATCCCCGCTTCAGAGCAAAA |
| qMHV-R | GGCTGTTTGCAACCCAGAAG |
| qPDCoV-F | ttcgtggcaatggagttc |
| qPDCoV-R | ggatggaggaatcggagta |
| qPoRV-F | TATGACACCAGCAGTTGCGA |
| qPoRV-R | GTCCGCAAGCACAGATTCAC |
| Scaffold oligo | AAAAGCACCGACTCGGTGCCACTTTTTCAAGTTGATAACGGACTAGCCTTATTTTAACTTGCTATTTCTAGCTCTAAAAC |
| PQSmut-F1 | CAGGTTGATAATGGTTCAAGC |
| PQSmut-R1 | GTCAACTCTAAACCACCAGCCCACAACACAAA |
| PQSmut-F2 | CTGGTGGTTTAGAGTTGACAACTATGCGTTA |
| PQSmut-R2 | GTTACTAAATGTCTGCCACAG |
| PQSmut-sgRNA-a | GGATCCTAATACGACTCACTATAGGGGTTCAAGCATTAATCGCAGTTTTAGAGCTAGA |
| PQSmut-sgRNA-b | GGATCCTAATACGACTCACTATAGGGGACACCTATAATCTGTGGGTTTTAGAGCTAGA |
| GRP78-F | CTTCGGCGTGAGGTGGAAAA |
| GRP78-R | ACCGGAACAGATCCATGTTGA |
| GADD34-F | GTCAGGACCTGTGATCGCTT |
| GADD34-R | CCATGTGTCTGGGCGGTG |
| ATF4-F | CCAACAACGGCAAGGAGGATG |
| ATF4-R | GGGCATCAAAGTCGAACTCC |
| B2M-F | GCGGAATATAAGTGGAGGCGT |
| B2M-R | ATCTTTGGAGCACGCTGGATA |
| XBP1-F | CGGCCTTGTAGTTGAGAACCA |
| XBP1-R | TCACTCCATTCCCCTTGGCTT |

**Table S2. Prediction of anti-PEDV activity for shared porphyrin ring compounds.**

| Name | Activity Score | Predicted Rank | Rank Percentile |
| --- | --- | --- | --- |
| TPP(4-Cl)4 | 0.920242 | 1 | 0% |
| Meso-tetra(o-dichlorophenyl)porphine | 0.911284 | 2 | 0% |
| Tetraphenylporphyrin | 0.908312 | 3 | 0% |
| meso-Tetratolylporphyrin-Ni (II) | 0.906092 | 4 | 0% |
| Tetra(p-tolyl) porphyrin | 0.904981 | 5 | 0% |
| Cobalt tetramethoxyphenylporphyrin | 0.882508 | 7 | 0% |
| TPPS4 | 0.874951 | 9 | 0% |
| CHlorin e6 trimethyl ester | 0.874635 | 10 | 0% |
| Vitamorell | 0.796466 | 28 | 0% |
| Sodium iron chlorophyllin | 0.792908 | 32 | 0% |
| Protoporphyrin IX | 0.786013 | 35 | 1% |
| TPAPP | 0.763159 | 46 | 1% |
| Chlorin E6 trisodium salt | 0.746635 | 58 | 1% |
| Protoporphyrin IX dimethyl ester | 0.592519 | 336 | 5% |
| Tetra(4-cyanophenyl) porphyrin | 0.553168 | 500 | 7% |
| ZnPP | 0.443764 | 1103 | 16% |
| Chlorophyllin | 0.2 | 3917 | 58% |
| TMPyP4 tosylate | 0.2 | 3918 | 58% |
| Copper perfluorophthalocyanine | 0.2 | 3919 | 58% |
